# The Interplay Between Beta-Amyloid 1–42 (Aβ_1–42_)-Induced Hippocampal Inflammatory Response, p-tau, Vascular Pathology, and Their Synergistic Contributions to Neuronal Death and Behavioral Deficits

**DOI:** 10.3389/fnmol.2020.552073

**Published:** 2020-11-02

**Authors:** Beatriz Calvo-Flores Guzmán, Tessa Elizabeth Chaffey, Thulani Hansika Palpagama, Sarah Waters, Jordi Boix, Warren Perry Tate, Katie Peppercorn, Michael Dragunow, Henry John Waldvogel, Richard Lewis Maxwell Faull, Andrea Kwakowsky

**Affiliations:** ^1^Centre for Brain Research, Department of Anatomy and Medical Imaging, Faculty of Medical and Health Sciences, University of Auckland, Auckland, New Zealand; ^2^Centre for Brain Research, NeuroDiscovery Behavioural Unit, Faculty of Medical and Health Sciences, University of Auckland, Auckland, New Zealand; ^3^Department of Biochemistry, University of Otago, Dunedin, New Zealand; ^4^Centre for Brain Research, Department of Pharmacology, Faculty of Medical and Health Sciences, University of Auckland, Auckland, New Zealand

**Keywords:** Alzheiemer’s disease, β-amyloid, tau phosphorylation, cognition, neuroinflamamation

## Abstract

Alzheimer’s disease (AD), the most common chronic neurodegenerative disorder, has complex neuropathology. The principal neuropathological hallmarks of the disease are the deposition of extracellular β-amyloid (Aβ) plaques and neurofibrillary tangles (NFTs) comprised of hyperphosphorylated tau (p-tau) protein. These changes occur with neuroinflammation, a compromised blood-brain barrier (BBB) integrity, and neuronal synaptic dysfunction, all of which ultimately lead to neuronal cell loss and cognitive deficits in AD. Aβ_1–42_ was stereotaxically administered bilaterally into the CA1 region of the hippocampi of 18-month-old male C57BL/6 mice. This study aimed to characterize, utilizing immunohistochemistry and behavioral testing, the spatial and temporal effects of Aβ_1–42_ on a broad set of parameters characteristic of AD: p-tau, neuroinflammation, vascular pathology, pyramidal cell survival, and behavior. Three days after Aβ_1–42_ injection and before significant neuronal cell loss was detected, acute neuroinflammatory and vascular responses were observed. These responses included the up-regulation of glial fibrillary acidic protein (GFAP), cell adhesion molecule-1 (PECAM-1, also known as CD31), fibrinogen labeling, and an increased number of activated astrocytes and microglia in the CA1 region of the hippocampus. From day 7, there was significant pyramidal cell loss in the CA1 region of the hippocampus, and by 30 days, significant localized up-regulation of p-tau, GFAP, Iba-1, CD31, and alpha-smooth muscle actin (α-SMA) in the Aβ_1–42_-injected mice compared with controls. These molecular changes in Aβ_1–42_-injected mice were accompanied by cognitive deterioration, as demonstrated by long-term spatial memory impairment. This study is reporting a comprehensive examination of a complex set of parameters associated with intrahippocampal administration of Aβ_1–42_ in mice, their spatiotemporal interactions and combined contribution to the disease progression. We show that a single Aβ injection can reproduce aspects of the inflammatory, vascular, and p-tau induced pathology occurring in the AD human brain that lead to cognitive deficits.

## Introduction

Alzheimer’s disease (AD) is a progressive neurodegenerative disorder characterized by a widespread loss of neuronal synapses and spines, the presence of intracellular neurofibrillary tangles (NFTs), and extracellular β-amyloid (Aβ) plaques (Huang and Mucke, 2012; Brun and Englun, 1981). During the last two decades, these features have been incorporated into the amyloid cascade hypothesis (Karran et al., 2011). Today, the successive downstream events occurring as a result of Aβ aggregation and spread represent a key thread of the hypotheses to explain the pathology observed in AD (Hardy and Selkoe, 2002). Despite a large number of clinical trials, there is still currently no effective treatment to prevent, significantly delay, or ameliorate the debilitating symptoms of AD.

This is largely due to our limited understanding of the connecting factors underlying the disease, as well as the poor translation of promising treatment options derived from animal models to human clinical trials (Franco and Cedazo-Minguez, 2014; Souchet et al., 2018). With the prevalence of AD increasing alarmingly, it is crucial to develop animal models that more closely mimic the pathological and clinical symptoms of human AD. Importantly, such a model has to be well characterized to document its limitations (Drummond and Wisniewski, 2017) and to determine whether it might effectively support drug screening for the development of novel and effective treatments for AD.

A rising concentration of Aβ in the brain is a critical factor in the development of late-onset sporadic AD, playing a key role in triggering the “amyloid cascade.” Although transgenic models, such as amyloid protein precursor (APP)- and presenilin-1 (PS-1)-overexpressing models are known to be useful in the study of genetic aspects of early-onset AD (Ohno et al., 2004, 2007), the late-onset sporadic form of the disease, that accounts for approximately 90% of all cases (Bekris et al., 2010), requires unique approaches to model this form of AD. Growing evidence indicates that Aβ-injected rodent models of AD might closely mimic the main neuropathological symptoms present in AD patients when concentrations of Aβ are increasing (Puzzo et al., 2014; Kwakowsky et al., 2016; Facchinetti et al., 2018; Baluchnejadmojara et al., 2019; Mudò et al., 2019; Yeung et al., 2020a,b). With this model, many aspects of AD-related pathology post-Aβ-injection and their link to the cognitive deficits are not fully characterized. Some of the key events occurring in the AD brain as a consequence of increased Aβ load include neuroinflammation and the disruption of the blood-brain barrier (BBB), both of which contribute to the progression of the disease (Soto-Rojas et al., 2015). For example, the abnormal accumulation and spread of Aβ can lead to localized inflammation involving reactive astrocytes with increased glial fibrillary acidic protein (GFAP) expression (Kamphuis et al., 2012), as well as the activation of the microglia that surround Aβ plaques early in the disease (Navarro et al., 2018). In response to high Aβ load and as part of the initiation of the inflammatory process, both astrocytes and microglia likely up-regulate their expression of a surface receptor for the monocyte chemotactic protein-1 (MCP-1), a β-chemokine involved in inflammation that regulates infiltration/migration of macrophages and microglia (Conductier et al., 2010). It has been recently shown that both the severity of AD (Lee et al., 2018b) and the associated memory deficits (Bettcher et al., 2019) correlate with increased plasma levels of MCP-1 in affected humans. Despite evidence supporting microgliosis as a direct response to Aβ load in the AD brain, and/or an indirect process underlying neuronal death (Marín-Teva et al., 2011), the precise role of microglia in the progression of the disease and response to Aβ load has not been fully elucidated. Interferon gamma-induced protein 10 (IP-10) is a pro-inflammatory chemokine that plays a key role in the inflammatory process and is highly expressed in astrocytes in the AD brain (Xia et al., 2000). Furthermore, increasing severity of AD is associated with increased levels of IP-10 (Leung et al., 2013). Aβ load also correlates with the level of astrocytic GFAP expression in 3xTg-AD mice and AD patients (Wyssenbach et al., 2016).

There is increasing evidence to suggest that the BBB is affected by Aβ deposition, which might contribute to its leakage and dysfunction (Erickson and Banks, 2013). The basic functional unit of the BBB is the neurovascular unit (NVU). In capillaries, this consists of endothelial cells connected by tight junctions, pericytes, astrocyte end-feets and extracellular matrix components of the basement membrane (Muoio et al., 2014). Most components of the BBB have been found to contribute to vascular dysfunction in AD (Govindpani et al., 2019, 2020).

A triggering event of AD pathology might be the accumulation of Aβ in the vasculature, leading to a vicious cycle of Aβ aggregation and BBB dysfunction (Govindpani et al., 2020). In this regard, the leakage of fibrinogen, a protein excluded from the brain by the BBB, has been implicated in AD vascular pathology. The fibrinogen-Aβ interaction causes aggregation of fibrinogen and significantly increased BBB permeability through the down-regulation of endothelial tight junction proteins (Cortes-Canteli et al., 2012). Aβ deposition in the vasculature also affects endothelial cell function in the NVU. For instance, endothelial cell adhesion molecules PECAM-1 and ICAM-1 (also known as CD31 and CD54, respectively) play a role in regulating interactions between leukocytes and the endothelium and are involved in the AD pathology through their contribution to the inflammatory process within blood vessels (Wennström and Nielsen, 2012). In the same way, the up-regulation of α-SMA, which plays a role in the contraction of the vessels, might be a compensatory mechanism in late-stage of the AD pathology in response to early vascular disruption in the BBB (Hutter-Schmid and Humpel, 2016).

NFT load has also been found to correlate with the severity of AD (Arriagada et al., 1992). The pathological effect of p-tau in AD may be due to the loss of function of normal tau together with the toxic gain of function of p-tau, which ultimately leads to impaired axonal transport and compromises cell function and homeostasis (Pritchard et al., 2011; Kruger and Mandelkow, 2015; Huber et al., 2017). Neuroinflammation (Lee et al., 2008), compromised BBB function (Nelson et al., 2016) and p-tau accumulation (Huber et al., 2017) are inferred to be the main pathological mechanisms underlying cognitive impairment in AD (Zempel and Mandelkow, 2014; van de Haar et al., 2016; Bettcher et al., 2019). These mechanisms likely promote extensive degeneration of excitatory pathways in brain areas such as the cerebral cortex and hippocampus.

Excitatory N-methyl-D-aspartate receptors are known to mediate Aβ_1–42_-induced excitotoxicity during AD (Liu et al., 2008). Nevertheless, inhibitory pathway disruptions are also well identified in the AD brain (Rissman and Mobley, 2011; Limon et al., 2012; Fuhrer et al., 2017; Govindpani et al., 2017; Kwakowsky et al., 2018). It has been shown that Aβ_1–42_ can increase ambient γ-aminobutyric acid (GABA) concentrations (Kwakowsky et al., 2020; Vinnakota et al., 2020). Recent evidence postulates that this increased ambient GABA level might activate extrasynaptic GABA_A_ receptors (GABA_A_Rs) in the hippocampus leading to a chronic depolarizing block through increased tonic inhibition in this area. This results in neural dystrophy and contributes to cognitive decline (Marczynski, 1998; Calvo-Flores Guzmán et al., 2020; Yeung et al., 2020b).

Despite the extensive research in this area, both utilizing *in vitro* and *in vivo* transgenic animal models as well as AD patients, both the definition and the understanding of the disease pathophysiology are far from precise (Govindpani et al., 2017, 2019; Boche and Nicoll, 2020; Harris et al., 2020). Consequently there is a need for a better understanding of the mechanisms underlying Aβ-induced molecular, cellular, and behavioral changes. Some AD rodent models injected with different types of Aβ fragments intraventricularly, or to specific brain regions, have been used to model the disease (Cetin et al., 2013; Faucher et al., 2016; Kwakowsky et al., 2016; Nicole et al., 2016; Schmid et al., 2017). However, it has been shown that the Aβ_1–42_ peptide introduced to neurons *in vitro* and *in vivo* is considerably more neurotoxic than that generated in the AD brain (Klein et al., 1999). This may be because it contains misfolded Aβ_1–42_ and thereby already has enhanced aggregation (Hillen, 2019).

The goal of this study is to elucidate the spatiotemporal progression of Aβ_1–42_-induced pathology and its connection to the resulting synergies of the molecular and cellular changes in p-tau, GFAP, IBA-1, IP-10, MCP-1, ICAM-1, αSMA, CD31, and fibrinogen in the hippocampus, and the resulting behavioral deficits. We have examined the local layer-specific changes of p-tau, and these neuroinflammatory and vascular markers in the CA1 region of the mouse hippocampus using immunohistochemistry and have shown they lead to severe impairment of long-term spatial memory.

## Materials and Methods

### Animals and Brain Tissue Preparation

All experiments were approved and performed following the regulations of the University of Otago and the University of Auckland. All mice were bred and housed at the Hercus Taieri Resource Unit, the University of Otago and Vernon Jansen Unit, University of Auckland, under 12-h reverse light-cycle conditions (lights on at 8 PM), with *ad libitum* access to food and water. All experiments were conducted following the National Animal Ethics Advisory Committee guidelines and with the approval of the institutional animal ethics committee of the University of Otago and the University of Auckland. All experiments were performed on old (18 months; immunohistochemistry, *n* = 6/group; behavioral testing, *n* = 12/group) C57BL/6 wild-type male mice.

### Aβ_**1–42**_ Preparation

Aβ_1–42_ is routinely produced as a recombinant protein fused with maltose-binding protein (MBP), with a proteolytic cleavage site for Factor X protease between the two segments based on as used in (Kwakowsky et al., 2020, 2016; Calvo-Flores Guzmán et al., 2020; Yeung et al., 2020a,b). This strategy utilizes the solubilizing character of the MBP, a product of the MalE gene, to ensure the expression of the soluble fusion protein at a high concentration in *Escherichia coli*. After the bacterial expression of this recombinant fusion protein, the product was purified on an amylose column to which the MBP segment of the protein binds. The affinity selected fusion protein was eluted from the resin with maltose and concentrated by ammonium sulfate precipitation. The carrier MBP was then cleaved off the fusion protein by Factor X protease, and the released Aβ_1–42_ was isolated and further purified by hydrophobic chromatography with 0–50% v/v acetonitrile/0.1% v/v Trifluoroacetic acid (TFA), using fast protein liquid chromatography (FPLC). The fractions containing pure Aβ_1–42_ were detected immunologically with an antibody against residues 17–24 of Aβ_1–42_ and lyophilized to remove the solvent. Mass spectrometry was used to confirm the expected molecular ion for the desired product. The concentration of the protein fragment has been determined by bicinchoninic acid assay at 60°C for 30 min. Before intra-hippocampal injection of this product, we diluted the prepared monomer in artificial cerebrospinal fluid [ACSF: 147 mM Na^+^, 3.5 mM K^+^, 2 mM Ca^2+^, 1 mM Mg^2+^ (pH 7.3)] and “aged” the solution at 37°C for 48 h to facilitate the formation of soluble aggregates, which was confirmed by SDS/PAGE and by non-dissociating PAGE (Yeung et al., 2020a). The optimal incubation time required to produce the highly toxic oligomers is 48–120 h depending on the preparation (Kwakowsky et al., 2016; Calvo-Flores Guzmán et al., 2020; Yeung et al., 2020a).

### Aβ_**1–42**_ Stereotaxic Injection

Mice were anesthetized by subcutaneous injection of 75 mg/kg ketamine and 1 mg/kg domitor. Bilateral coordinates for stereotaxic Aβ_1–42_ injection at three depths (antero-posterior, −2.0 mm; medio-lateral, ±1.3 mm; dorso-ventral, −1.8, 2.0, and 2.2 mm from the dura) within the CA1 region of the hippocampus were determined relative to the bregma according to the Paxinos and Franklin’s mouse brain atlas (Yeung et al., 2020a,b). Stereotaxic bilateral administration of 1 μl of 20 μM neurotoxic Aβ_1–42_ or scrambled Aβ_1–42_ (scrAβ_1–42_, AS-25382, AnaSpec) or artificial cerebrospinal fluid (ACSF, used as a vehicle) into the CA1 region was performed at a rate of 0.1 μl/min. The mice in this study were categorized into four groups: naïve control (NC), ACSF, scrAβ_1–42_, and Aβ_1–42_ injected groups. Naïve control animals did not undergo any surgical procedures.

### Behavioral Testing

Behavioral testing was performed to elucidate the effects of Aβ_1–42_ on the cognitive performance of the mice. Specific behavioral tests were used to target different types of hippocampal-dependent memories: (i) long-term spatial memory with the novel object alteration (NOA), novel object recognition (NOR) test, and the Morris water maze (MWM) tests; and (ii) short-term spatial memory with the Y-maze test (YM), as well as short-term non-spatial memory with the passive avoidance test. The O-maze (OM) test was used as a measurement of the anxiety levels of the mice. All behavioral tests were started at 9 am, and analysis was performed using the tracking image analyzer system EthoVision XT 9 (Noldus).

### Novel Object Alteration Test

The NOA test was performed to evaluate long-term working memory 7–8 days after injection. The test was performed in a square arena that was surrounded by non-transparent plexiglass walls (25 cm × 29 cm × 25 cm). Each mouse was placed in the arena individually and given 10 min to habituate to the environment. Next, two identical objects were introduced in the arena at designated locations, and the mice were given 5 min to interact with and explore the objects. Following this, each mouse was returned to its cage. The following day (24 h later), one of the identical objects was placed in a new location, and the behavior of the mice was recorded over a 5 min testing period. The testing apparatus was cleaned between animals with 5% acetic acid to minimize olfactory cues. The discrimination ratio (DR) for a novel over a familiar object was calculated as follows: time spent near the object at the new position minus the time spent near the object at the old position, divided by time spent near the object at the new position plus the time spent near the object at the old position.

### Novel Object Recognition Test

The NOR test to evaluate long-term recognition memory was performed 11 days after injection. in the same arena as the NOA. During the first 10 min session on day 1, the animal was free to explore the arena, and during the second 5 min session, the animal was able to explore two identical objects. On day 2, one of the objects was replaced by a novel, unfamiliar object, and animal behavior was recorded for 5 min.

The amount of time spent to explore the new object is considered as an index of recognition memory. The DR for a novel over a familiar object was calculated as follows: time spent near the new object minus the time spent near the old object, divided by time spent near the new object plus the time spent near the old object.

### Y-Maze Test

Spontaneous exploration and responsiveness to novel environments and short-term spatial memory functions were evaluated with the YM test 15 days post-injection. The apparatus used for the YM study was constructed out of plexiglass with the three arms of the maze positioned at a 120° angle relative to each other. Each arm is identical (52 cm × 12.5 cm); however, different spatial cues were placed in each arm. The start arm for each experiment was chosen randomly: each mouse was placed in the YM environment on two occasions that were separated by a 2 min interval. During the first 5 min trial, one of the three arms was randomly blocked. In the second trial, all the arms were opened for exploration; the total amount of time the mouse took to explore each arm was recorded for 3 min. During the inter-trial interval (2 min), the animal was returned to its home cage and the maze was cleaned. The alternation percentage was calculated as the percentage of the ratio of actual to possible alternations. An index of the time spent in the new, previously unexplored arm as opposed to the familiar explored arm was used to assess any behavioral differences between each group and was calculated as follows: time spent in the new arm minus time spent in the old arm, divided by time spent in the new arm plus time spent in the old arm.

### Morris Water Maze Test

The MWM, a reliable test of spatial memory and hippocampal-dependent learning, was performed at 20 days post-injection (D’Hooge and De Deyn, 2001). The MWM apparatus comprised a circular black tank (diameter, 130 cm; height, 130 cm) filled with tap water and powdered non-fat milk that was added to the tank before the experiment. A constant temperature of 20°C was maintained during the test. A circular escape platform of ~10 cm diameter and several navigation cues were used to provide spatial orientation for the mice. The starting position of every mouse was assigned randomly. The location of the hidden platform was kept constant (except on the last day of the experiment). If the mouse did not find the hidden platform within 60 s, the animal was guided to the platform for 10 s before being returned to the cage. Spatial learning was tested across four repeat trials over the following 4 days. Between trials, mice were dried with a towel and placed in their cages, located over heating blankets. On the fifth day in each trial, the escape platform was removed, and the time taken to reach the platform quadrant, time spent in the platform quadrant, and distance traveled to reach the platform for each animal were assessed.

### Passive Avoidance Test

The passive avoidance test was performed 27 days post-injection. This associative learning task was conducted in a two-compartment box made of one bright compartment and one dark compartment (16 cm × 18 cm). During habituation, the mouse was placed in the bright compartment, and the mouse gained access to the dark compartment. When the mouse entered the dark compartment the door was closed, and the mouse was briefly administered a 0.3-mA electric shock on the foot for 2 s as an aversive stimulus. After 30 s the animal was returned to its home cage. Three hours later, the animal was returned to the bright compartment with the sliding door open. The animal now had the option to avoid or enter the dark compartment. The latency period before the mouse entered the dark compartment was measured.

### O-Maze Test

The OM test was performed at 17 days post-injection to assess anxiety-like behaviors. The OM apparatus consisted of a circular maze (40 cm diameter) with two protected (closed) arms, where the mice usually feel safer, and two unprotected (open) arms. Each mouse was randomly placed in one of the closed arms and the behavior was recorded for 5 min. The total time spent by each mouse in the closed and protected arms was measured. Anxiety-like behavior was estimated based on the total time spent in the closed arms of the apparatus, indicating the amount of time spent avoiding the new environment.

### Western Blotting

**Figure 1:**
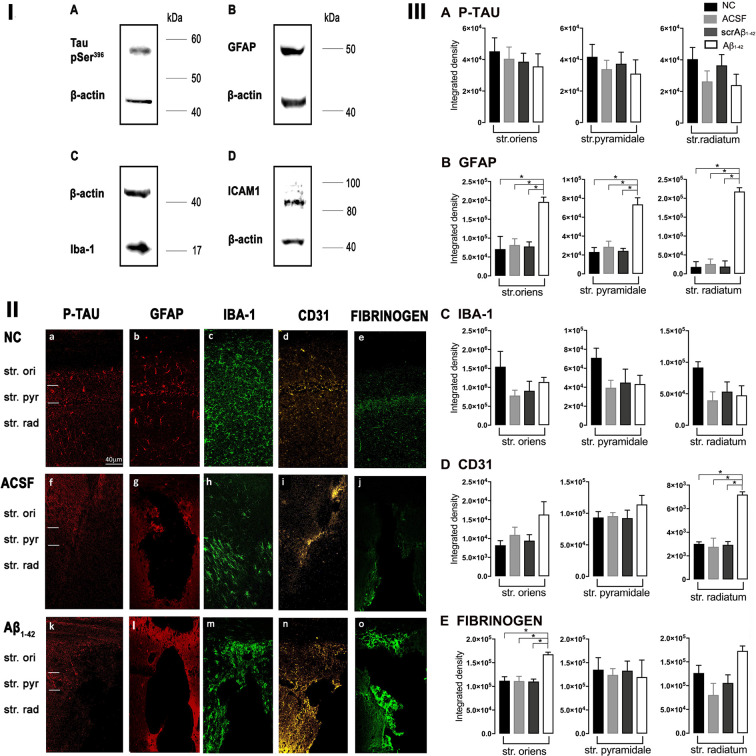
**Panel I:** Western blot against mouse brain protein homogenates probed with p-tau **(A)**, glial fibrillary acidic protein (GFAP; **B**), Iba-1 **(C)**, and ICAM-1 **(D)**. Observed band sizes: p-tau ~58 kDA, GFAP ~50 kDA, Iba-1 ~17 kDA and ICAM-1 ~85 kDA. **Panel II:** Representative images showing immunolabeling of p-tau, GFAP, Iba-1, CD31, and fibrinogen at the injection site, 3 days post-injection in NC **(a–e)**, ACSF- **(f–j)**, and Aβ_1–42_-injected **(k–o)** mice. Scale bar (40 μm). **Panel III:** Graphs showing quantification at the injection site in the CA1 hippocampal region of p-tau, GFAP, Iba-1, CD31, and fibrinogen immunolabeling density, 3 days post-injection in NC, ACSF-, scrAβ_1–42_- and Aβ_1–42_-injected mice. Data expressed as mean ± SEM (Kruskal–Wallis test; *p < 0.05, *n* = 3–5).

The specificity of the antibodies used in this study had either been tested and reported previously (Yang et al., 2014; Llorian et al., 2016; Wang et al., 2016) or was examined using Western blotting (Figure 1I; using a method published previously; Palpagama et al., 2019a,b; Pandya et al., 2019). Mice were euthanized by cervical dislocation and the brains rapidly removed. The brain was cut in half separating the hemispheres on ice; the hippocampus was dissected from each hemisphere of the brain, freshly snap-frozen on dry ice, and stored at −80°C. Tissue was homogenized using a lysis buffer: 4% SDS, 50 mM Tris-HCL, 2 mM EDTA, pH 6.8 supplemented with 0.1% protease inhibitor cocktail (Sigma-Aldrich Co., Saint Louis, MO, USA: P8340) and with 1 mM phenylmethylsulfonyl fluoride (PMSF; Sigma–Aldrich Co., Saint Louis, MO, USA: P7626). A mixture of tissue and lysis buffer was then transferred to centrifuge tubes containing 0.5 mm glass beads. Tissue was homogenized using a Bullet Blender Tissue Homogeniser (Next Advance, Inc., Troy, NY, USA) at speed 8 for 4 min. Samples were then left on the ice to incubate for 1 h and centrifuged at 10,621 *g* for 10 min at 4°C. The supernatant was collected and stored at −20°C. Protein concentrations were determined using a detergent-compatible protein assay (DC Protein assay, 500-0116, Bio-Rad, Hercules, CA, USA), following the manufacturer’s instructions. Twenty μg of each protein extract was run on a gradient-polyacrylamide electrophoresis gel (NU PAGE 4–12% BT 1.5, Life Technologies, CA, USA) at 200 V for 45 min using a Thermo Fisher Mini Gel Tank and transferred onto nitrocellulose membranes using the XCell Blot Module (Invitrogen, Waverley, VIC, Australia) at 30 V for 90 min. Membranes were washed in Tris-HCl buffered saline (TBS; pH 7.6) with 0.1% Tween (TBST) and blocked for 30 min at room temperature (RT) with Odyssey blocking buffer (LI-COR Biosciences, USA). The membranes were incubated with primary antibodies (Table 1) overnight at 4°C in TBST with 4% BSA (BSA-TBST). The following day, after 3 × 10 min washes in TBST, membranes were incubated at RT for 1 h with an appropriate IRDye (1:10,000, goat anti-rabbit IRDye 680RD, 926-68071, RRID:AB_10956166; goat anti-mouse IRDye 800CW, 926-32210, RRID:AB_621842; donkey anti-goat IRDye 800CW, 926-32214, RRID:AB_621846; LI-COR Biosciences, Lincoln, NE, USA) secondary antibody diluted in 4% BSA-TBST. Detection of immunoreactive bands was performed using the Odyssey Infrared Imaging System (LI-COR Biosciences, USA).

**Table 1:** Primary antibodies used in this study.

Antigen	Immunogen	Source, host species, catalog number	Dilution for WB	Dilution for IHC
Anti- Tau pSer396	MAPT(human) mapping to 17q21.31; Mapt(mouse) mapping to 11 E1	Santa Cruz, polyclonal rabbit, sc-101815	1/200	1/100
GFAP cocktail	Equal concentrations of all three monoclonal antibodies (4A11, 1B4, 2E1) that specifically recognize GFAP	BD Biosciences, monoclonal mouse, 556330	1/10,000	1/5,000
Iba-1	A synthetic peptide corresponding to human Iba-1 amino acid 135–147 (C-terminal)	Abcam, polyclonal goat, ab5076	1/500	1/1,000	
IP-10	Highly pure (>98%) recombinant hIP 10	Abcam, polyclonal rabbit, ab9807	-	1/100	
MCP-1	A recombinant fragment corresponding to human MCP-1	Abcam, polyclonal rabbit, ab9669	-	1/100	
α-SMA	N-terminal synthetic decapeptide of α-SMA coupled to keyhole limpet hemocyanin (KLH)	Dako, monoclonal, mouse, M0851	-	1/10	
ICAM-1 (CD54)	ICAM-1 (human) mapping too 19p13.2; ICAM-1 (mouse) mapping to 9A3	Santa Cruz, monoclonal mouse, sc-107	1/50	1/50	
Fibrinogen	Fibrinogen isolated from human plasma	Dako, polyclonal rabbit, A0080	-	1/500	
PECAM-1 (CD31)	Human extracellular domain 1	Dako, monoclonal mouse, M0823	-	1/50	
NeuN	Purified cell nuclei from mouse brain	Millipore, monoclonal mouse, MAB377	-	1/1,000	

*IHC, immunohistochemistry; WB, Western blotting*.

### Free-Floating Fluorescence Immunohistochemistry

Mice were deeply anesthetized with 75 mg/kg ketamine and 1 mg/kg domitor and perfused transcardially with 20 ml of ice-cold 4% paraformaldehyde in phosphate buffer (pH 7.6). Brains were removed and postfixed in 4% paraformaldehyde solution for 2 h at RT and then incubated in 30% sucrose in TBS (pH 7.6; 0.05 M Tris-HCl, 0.15 M NaCl) overnight at 4°C. Hippocampal coronal sections (30 μm) were cut on a freezing microtome (Microm International GmbH, Walldorf, Germany) and collected in TBS.

Free-floating fluorescent immunohistochemistry was performed using the method described by Kwakowsky et al. (2016). Briefly, hippocampal sections were washed 3 × 10 min with TBS and incubated in 0.05 M TBS/0.3% Triton/0.25% BSA (TTB)/1% goat serum for 1 h at RT. Sections were then incubated with primary antibodies (Table 1) diluted in TTB for 72 h at 4°C on two hippocampal sections from each group. Following 3 × 10 min washes in TBS, sections were incubated in secondary antibodies, goat anti-rabbit Alexa Fluor 647 (1:500, A21245, RRID:AB_141775; Invitrogen, Carlsbad, CA, USA), goat anti-mouse Alexa Fluor 488 (1:500, A11029, RRID: AB_138404; Invitrogen), goat anti-mouse Alexa Fluor 647 (1:500, A21236, RRID:AB_141725; Invitrogen), goat anti-rabbit Alexa Fluor 488 (1:500, A11034, RRID:AB_2576217; Invitrogen), donkey anti-goat Alexa Fluor 647 (1:500, A21447, RRID:AB_141844; Invitrogen) and donkey anti-rabbit Alexa Fluor 488 (1:500, A21206, RRID:AB_141708; Invitrogen) diluted in TTB for 2 h at RT. Finally, the sections were incubated in Hoechst nuclear counterstain (1/10,000 in TTB) for 15 min at RT.

Stained sections were examined under a Zeiss LSM 710 confocal laser-scanning microscope (Carl Zeiss, Jena, Germany). The layer-specific labeling of each marker within the CA1 region of the hippocampus was locally analyzed at two specific sites (injection site or needle track, 0–50 μm from the needle track, and adjacent to injection site, 50–500 μm from the needle track) using ImageJ software (U. S. National Institutes of Health, Bethesda, MD, USA). After background subtraction and greyscale threshold determination, density measurements were performed for each marker from a defined area of interest measuring 22,748 μm^2^ at the injection site and 152,132 μm^2^ in each analyzed layer [stratum (str.) oriens, str. pyramidale, str. radiatum] at a location adjacent to the injection site. Particle count and area coverage measurements were conducted using this protocol on the entire field of view of acquired images. Manual counting was performed to determine the number of primary astrocytic and microglial branches. The percentage area coverage by large particles is an indicator of area coverage by activated cells, set at threshold >150 pixels. The percentage area coverage by small particles is the measure of the area covered by the astrocyte and microglia processes. Cells with activated morphology tend to be larger, with more primary branches and an increased number of smaller processes (Glenn et al., 1992; Wilhelmsson et al., 2006; Boche et al., 2013; Palpagama et al., 2019b). The experimenter was blinded to the experimental groupings to eliminate any bias during the experiment, including during image acquisition and analysis. To assess the extent of pyramidal cell loss in the str. pyramidale of the CA1 region of the hippocampus post-Aβ_1–42_ injection, the number of NeuN-positive pyramidal neurons was counted in a 10,296 μm^2^ area of the str. pyramidale of the CA1 region. Sections in which the needle track was detected were used for analysis. Two sections were counted per animal (*n* = 6 in each group) and the results are presented as the number of NeuN-positive pyramidal neurons in the region of interest. Sections with NeuN labeling were examined under a Zeiss LSM 710 inverted confocal laser-scanning microscope (Carl Zeiss, Jena, Germany).

### Statistical Analysis

To examine the differences between groups, a Kruskal–Wallis test was conducted for the data obtained, using Graph-Pad Prism software version 8 (GraphPad software, RRID:SCR_002798) with a *p*-value of *p* ≤ 0.05 considered significant, as the data did not meet the assumptions of parametric tests assessed by the D’Agostino–Pearson omnibus and Brown–Forsythe tests. Correlation analysis was performed using Spearman’s test. Adobe Photoshop CC 2017 (Adobe Systems Software) was used to prepare the figures.

## Results

To assess cell layer-specific changes in tau pathology, density, and morphological changes in neuroinflammatory (GFAP, IBA-1, IP-10, MCP-1) and vascular markers (ICAM-1, αSMA, CD31, fibrinogen) within the CA1 hippocampal region, free-floating fluorescent immunohistochemistry was performed on tissues from NC, ACSF, scrAβ_1–42_- and Aβ_1–42_-injected mice. Quantification was performed 3 and 30 days post-Aβ_1–42_ injection at sites adjacent to the injection site, as well as at the injection site itself within the CA1 hippocampal region.

### Localized Aβ_**1–42**_-Induced Up-Regulation of GFAP and CD31 by 3 Days Post-Aβ_**1–42**_ Injection

**Figure 2:**
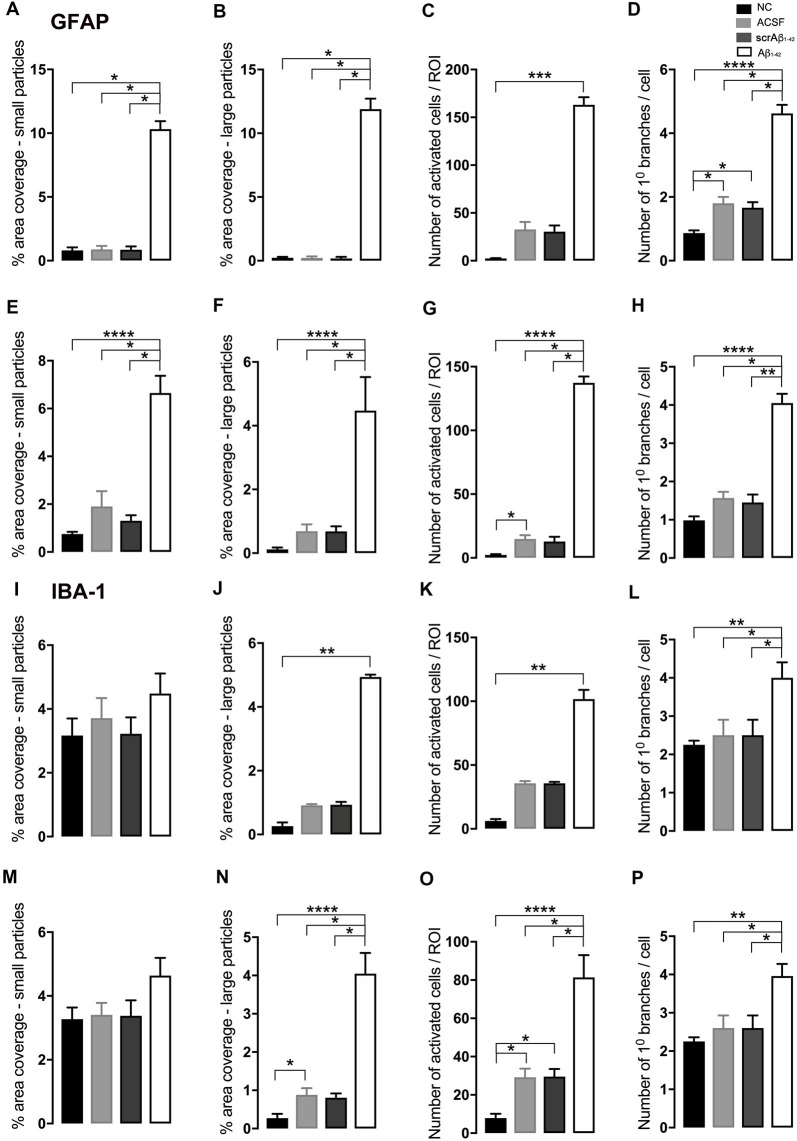
Graphs showing quantification of astrocytic and microglial morphology at the injection site **(A–D,I–L)** and a location adjacent to the injection site **(E–H,M–P)** in the CA1 hippocampal region 3 days post-injection in NC, ACSF-, scrAβ_1–42_- and Aβ_1–42_-injected mice. Data expressed as mean ± SEM (Kruskal–Wallis test; *p < 0.05; **p < 0.01, ***p < 0.001, ****p < 0.0001, *n* = 3–6).

At the injection site, Aβ_1–42_-injected mice did not display any significant cell loss in the str. pyramidale of the CA1 region at 3 days post-Aβ_1–42_ injection compared with control mice (Yeung et al., 2020a). Localized inflammatory- and vascular pathology-related changes were found in Aβ_1–42_-injected mice compared with ACSF-, scrAβ_1–42_-injected and NC mice (Figure 1, **panel II**). By contrast, p-tau did not show altered expression at the injection site 3 days post-Aβ_1–42_ injection (Figures 1IIa,f,k,IIIA). Aβ_1–42_-injected mice exhibited stronger immunostaining of GFAP, Iba-1, CD31, and fibrinogen (Figure 1II), markers in the CA1 region at the injection site in comparison with controls. There was an increased GFAP labeling density in comparison with NC, ACSF- and scrAβ_1–42_-injected mice within the str. oriens (*p* = 0.0415 vs. NC; *p* = 0.0499 vs. ACSF; *p* = 0.0315 vs. scrAβ_1–42_), str. pyramidale (*p* = 0.041 vs. NC; *p* = 0.0491 vs. ACSF; *p* = 0.032 vs. scrAβ_1–42_) and str. radiatum (*p* = 0.0416 vs. NC; *p* = 0.0421 vs. ACSF; *p* = 0.417 vs. scrAβ_1–42_) of the CA1 region (Figure 1IIIB). Aβ_1–42_-injected mice showed an increased area coverage by activated cells (*p* = 0.049 vs. NC; *p* = 0.049 vs. ACSF; *p* = 0.0235 vs. scrAβ_1–42_), a greater number of astrocytes with reactive morphology (*p* = 0.0003 vs. NC; *p* = 0.1838 vs. ACSF; *p* = 0.1554 vs. scrAβ_1–42_) with increased number of primary branches (*p* < 0.0001 vs. NC; *p* = 0.0499 vs. ACSF; *p* = 0.0398 vs. scrAβ_1–42_) and increased area coverage by astrocytic processes (*p* = 0.0315 vs. NC; *p* = 0.049 vs. ACSF; *p* = 0.0415 vs. scrAβ_1–42_; Figures 2A–D).

Aβ_1–42_-injected mice also showed an increase in numbers of reactive and dystrophic microglia, but the Iba-1 staining density did not increase (Figures 1IIm,IIIC, Figures 2K). There was also an increased area coverage by activated cells (*p* = 0.021 vs. NC; *p* = 0.9321 vs. ACSF; *p* = 0.99 vs. scrAβ_1–42_), a greater number of microglia with reactive morphology (*p* = 0.0053 vs. NC; *p* = 0.2170 vs. ACSF; *p* = 0.2170 vs. scrAβ_1–42_) with increased number of primary branches (*p* = 0.0044 vs. NC; *p* = 0.0399 vs. ACSF; *p* = 0.0399 vs. scrAβ_1–42_) but the area covered by microglial processes did not change (Figures 2I–L).

Aβ_1–42_-injected mice also showed an increased CD31 labeling density in comparison with NC, ACSF- and scrAβ_1–42_-injected mice within the str. radiatum (*p* = 0.0415 vs. NC; *p* = 0.049 vs. ACSF; *p* = 0.0315 vs. scrAβ_1–42_) of the CA1 region (Figure 1IIID). Fibrinogen displayed increased labeling in the brain parenchyma at the injection site compared with NC, ACSF- and scrAβ_1–42_-injected mice with a significant increase detected within the str. oriens (*p* = 0.042 vs. NC; *p* = 0.042 vs. ACSF; *p* = 0.042 vs. scrAβ_1–42_). A similar trend occurred in the str. radiatum but did not reach significance (Figures 1Ie,j,o,IIE).

**Figure 3:**
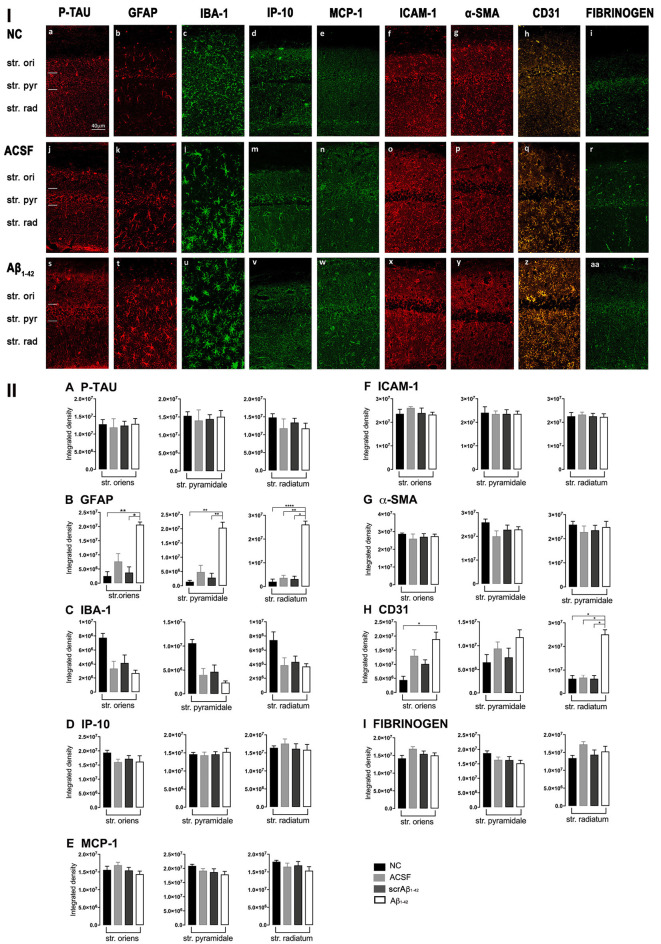
**Panel I:** Representative images showing immunolabeling of p-tau, GFAP, Iba-1, IP-10, MCP-1, ICAM-1, α-SMA, CD31, and fibrinogen at a location adjacent to the injection site, 3 days after injection in NC **(a–i)**, ACSF- **(j–r)** and Aβ_1–42_-injected **(s–aa)** mice. Scale bar (40 μm). **Panel II:** Graphs showing quantification at a location adjacent to the injection site in the CA1 hippocampal region of p-tau, GFAP, Iba-1, IP-10, MCP-1, ICAM-1, α-SMA, CD31, and fibrinogen **(A–I)** immunolabeling density, 3 days post-injection in NC, ACSF-, scrAβ_1–42_- and Aβ_1–42_-injected mice. Data expressed as mean ± SEM (Kruskal–Wallis test; *p < 0.05; **p < 0.01, ****p < 0.0001, *n* = 6).

Adjacent to the injection site, despite changes in the pattern of p-tau distribution between groups (Figure 3Ia,j,s), quantification of Tau pSer^396^ did not reveal any significant differences between NC, ACSF-, and scrAβ_1–42_-injected or Aβ_1–42_-injected mice in any layer of the CA1 region 3 days post-Aβ_1–42_ injection (Figure 3IIA). However, astrogliosis was revealed by a significant increase in the GFAP labeling intensity between NC, scrAβ_1–42_-injected and Aβ_1–42-_injected mice in the str. oriens (*p* = 0.0021 vs. NC; *p* = 0.0173 vs. scrAβ_1–42_), str. pyramidale (*p* = 0.036 vs. NC; *p* = 0.0076 vs. scrAβ_1–42_) and str. radiatum (*p* = 0.0003 vs. NC; *p* = 0.0071 vs. scrAβ_1–42_) of the CA1 region of the hippocampus (Figures 3Ib,k,t,IIB). Aβ_1–42_-injected mice also displayed an increased GFAP labeling in comparison with ACSF-injected mice within the str. radiatum (*p* = 0.0114) of the CA1 region (Figure 3IIB). Aβ_1–42_-injected mice showed a greater number of astrocytes with a highly reactive morphology indicated by numerous branching, elongated processes and hypertrophic cell bodies (Figure 3It). Aβ_1–42_-injected mice showed an increased area coverage by activated cells (*p* < 0.0001 vs. NC; *p* = 0.02 vs. ACSF; *p* = 0.0305 vs. scrAβ_1–42_), a greater number of astrocytes with reactive morphology (*p* < 0.0001 vs. NC; *p* = 0.0410 vs. ACSF; *p* = 0.0142 vs. scrAβ_1–42_) with increased number of primary branches (*p* < 0.0001 vs. NC; *p* = 0.0499 vs. ACSF; *p* = 0.0398 vs. scrAβ_1–42_) and increased area coverage by astrocytic processes (*p* < 0.0001 vs. NC; *p* = 0.0312 vs. ACSF; *p* = 0.0077 vs. scrAβ_1–42_; Figures 2E–H).

Despite Aβ_1–42_-injected mice displaying a greater increase in reactive and dystrophic microglia (Figure 3Iu), there were no significant differences in Iba-1 density among the groups at 3 days post-Aβ_1–42_ injection at locations adjacent to the injection site (Figure 3IIc). Aβ_1–42_-injected mice displayed an increased area coverage by activated microglia (*p* < 0.0001 vs. NC; *p* = 0.0275 vs. ACSF; *p* = 0.016 vs. scrAβ_1–42_), a greater number of microglia with reactive morphology (*p* < 0.0001 vs. NC; *p* = 0.049 vs. ACSF; *p* = 0.0475 vs. scrAβ_1–42_) with increased number of primary branches (*p* = 0.0012 vs. NC; *p* = 0.0286 vs. ACSF; *p* = 0.0286 vs. scrAβ_1–42_) but the area covered by microglial processes did not change (Figures 2M–P). Neither IP-10 nor MCP-1 levels, chemokines involved in inflammation, showed significant differences between the groups at 3 days post-Aβ_1–42_ injection (Figures 3Id,m,v,e,n,w,IID,E).

Concerning early vasculature disruption, labeling intensity was not significantly different among the groups in the markers ICAM-1 (Figures 3If,o,x,IIF), α-SMA (Figures 3Ig,p,y,IIG), or fibrinogen (Figures 3Ii,r,aa,III). However, Aβ_1–42_-injected mice showed early signs of vascular disruption with significantly up-regulated levels of the endothelial cell marker CD31 adjacent to the injection site in the str. oriens of the CA1 region of the hippocampus compared with NC (*p* = 0.0031), as well as in the str. radiatum of the CA1 region of the hippocampus compared with NC (*p* = 0.0131), ACSF-injected (*p* = 0.0387) and scrAβ_1–42_-injected mice (*p* = 0.0226; Figures 3Ih,q,z,IIH).

**Figure 4:**
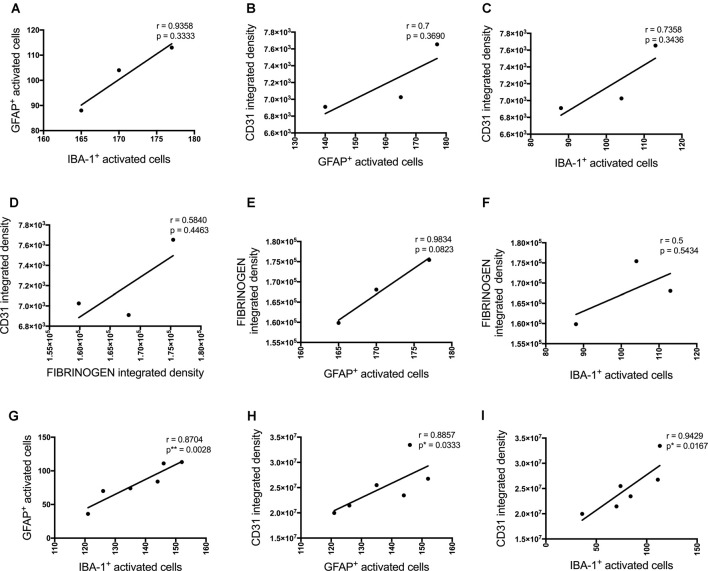
Correlation between the number of activated astrocytes and microglia and vascular markers at the injection site (**A–F**, *n* = 3) and a location adjacent to the injection site (**G–I**, *n* = 6) in the CA1 hippocampal region 3 days post-injection in NC, ACSF-, scrAβ_1–42_- and Aβ_1–42_-injected mice. This relationship is represented by a Spearman’s *r*-value (*p < 0.05, **p < 0.01).

These inflammatory (number of GFAP and IBA-1 positive activated cells) and vascular (CD31 and fibrinogen density) pathology markers showed multiple positive cross-correlations (Figure 4). For the data obtained at the injections site, these correlations were not statistically significant due to the limited number of sections available from the same animal to test all these markers (Figures 4A–F). However, significant positive correlations were observed for most of these markers adjacent to the injection site. The number of activated GFAP positive cells positively correlated with the number of activated microglia (*r* = 0.8704, *p* = 0.0028) and CD31 integrated density (*r* = 0.8857, *p* = 0.0333; Figures 3G,H). The number of IBA-1 positive activated cells also correlated with CD31 integrated density (*r* = 0.9429, *p* = 0.0167; Figure 4I).

### Localized Aβ_**1–42**_-Induced Pyramidal Cell Loss and Increase in p-tau, GFAP, Iba-1, CD31, and α-SMA by 30 Days Post-injection

**Figure 5:**
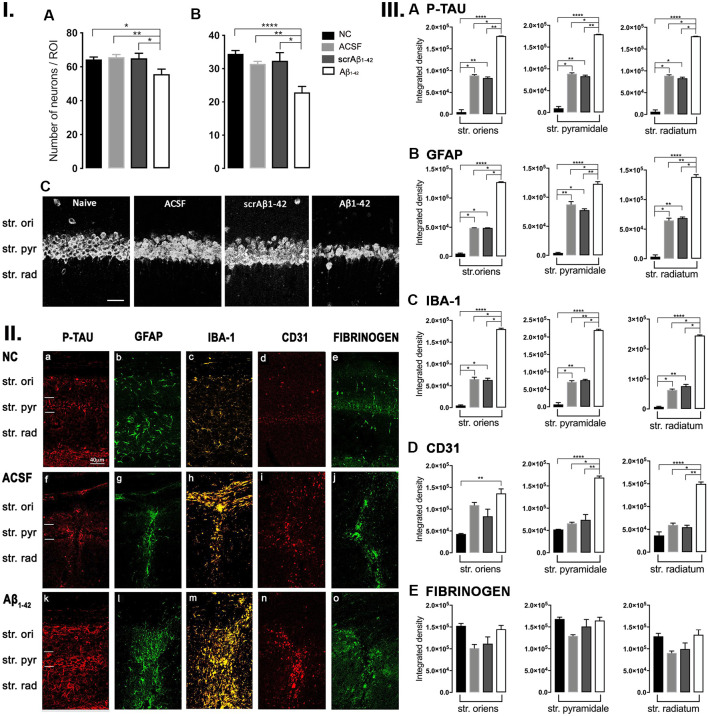
**Panel I:** Graphs showing the number of NeuN-positive pyramidal cells in the str. pyramidale of the CA1 hippocampal layer in NC, ACSF-, scrAβ_1–42_- and Aβ_1–42_-injected mice 7 days post-injection **(A)** and 30 days post-injection **(B)**. Representative images of the CA1 hippocampal subregion at a location adjacent to the injection site showing NeuN-positive pyramidal cells from the str. pyramidale in NC, ACSF-, scrAβ_1–42_- and Aβ_1–42_-injected mice 30 days post-injection **(C)**. **Panel II:** Representative images showing immunolabeling of p-tau, GFAP, Iba-1, CD31, and fibrinogen at the injection site 30 days post-injection in NC **(a–e)**, ACSF-injected **(f–j)**, and Aβ_1–42_-injected **(k–o)** mice. Scale bar (40 μm). **Panel III:** Graphs showing quantification at the injection site in the CA1 hippocampal region of p-tau, GFAP, Iba-1, CD31, and fibrinogen, 30 days post-injection in NC, ACSF-, scrAβ_1–42_- and Aβ_1–42_-injected mice. Data expressed as mean ± SEM (Kruskal–Wallis test; *p < 0.05; **p < 0.01, ****p < 0.0001, *n* = 6).

By day 30 post-Aβ_1–42_ injection the mice displayed significant neuronal cell loss in the str. pyramidale of the CA1 region of the hippocampus in comparison with the NC (23 ± 0.6 vs. 34.44 ± 1.02, *p* < 0.0001), ACSF-injected (23 ± 0.6 vs. 31.44 ± 0.75, *p* = 0.0412) and scrAβ_1–42_-injected mice (23 ± 0.6 vs. 32.38 ± 0.86, *p* = 0.0069; Figure 5IB). This 33%, 37% and 39% neuronal cell loss in the str. pyramidale of the CA1 region of the hippocampus in the Aβ_1–42_-injected mice in comparison with the NC mice, ACSF-, and scrAβ_1–42_-injected controls respectively (Figure 5IB) is indicative of Aβ_1–42_-induced neurotoxicity, as well as the long-lasting impact of a single bilateral intra-hippocampal injection of Aβ_1–42_. Visualization of NeuN-positive pyramidal cells in the str. pyramidale of the CA1 hippocampal region of Aβ_1–42_-injected mice demonstrated that apart from the considerable amount of cell loss, the remaining pyramidal cells had an irregular shape in Aβ_1–42_-injected mice (Figure 5IC). We have also found a significant 12%, 15% and 13% pyramidal cell loss at 7 days after Aβ_1–42_ injection in the CA1 hippocampal region of the mice compared to NC (56 ± 1.2 vs. 64 ± 1.34, *p* = 0.0458), ACSF- (56 ± 1.2 vs. 66 ± 1.59, *p* = 0.0164) and scrAβ_1–42_-injected mice, respectively (Figure 5IA).

At the injection site, significant localized tau hyperphosphorylation, inflammation, and vascular changes were found in Aβ_1–42_-injected mice 30 days post-injection compared with ACSF-injected and NC mice (Figures 5II,III). This conclusion was derived from localized Aβ_1–42_ injection-induced increase in p-tau, GFAP, and Iba-1 levels and the up-regulation of CD31 at the injection site (Figure 5IIk–m). By 30 days after the injection, p-tau immunoreactivity in the str. oriens, str. pyramidale and str. radiatum of the CA1 region of the hippocampus was significantly higher in the Aβ_1–42_-injected mice compared to NC (*p* < 0.0001), ACSF- (*p* = 0.0439; *p* = 0.044; *p* = 0.0123) and scrAβ_1–42_-injected mice (*p* = 0.0057; *p* = 0.0058; *p* = 0.0229; Figures 5IIa,f,k,IIIA). Aβ_1–42_-injected mice showed stronger p-tau immunoreactivity within the somatodendritic compartments of neurons in the str. pyramidale and along axonal processes extending from the str. pyramidale compared with control mice (Figure 5IIa,f,k).

**Figure 6:**
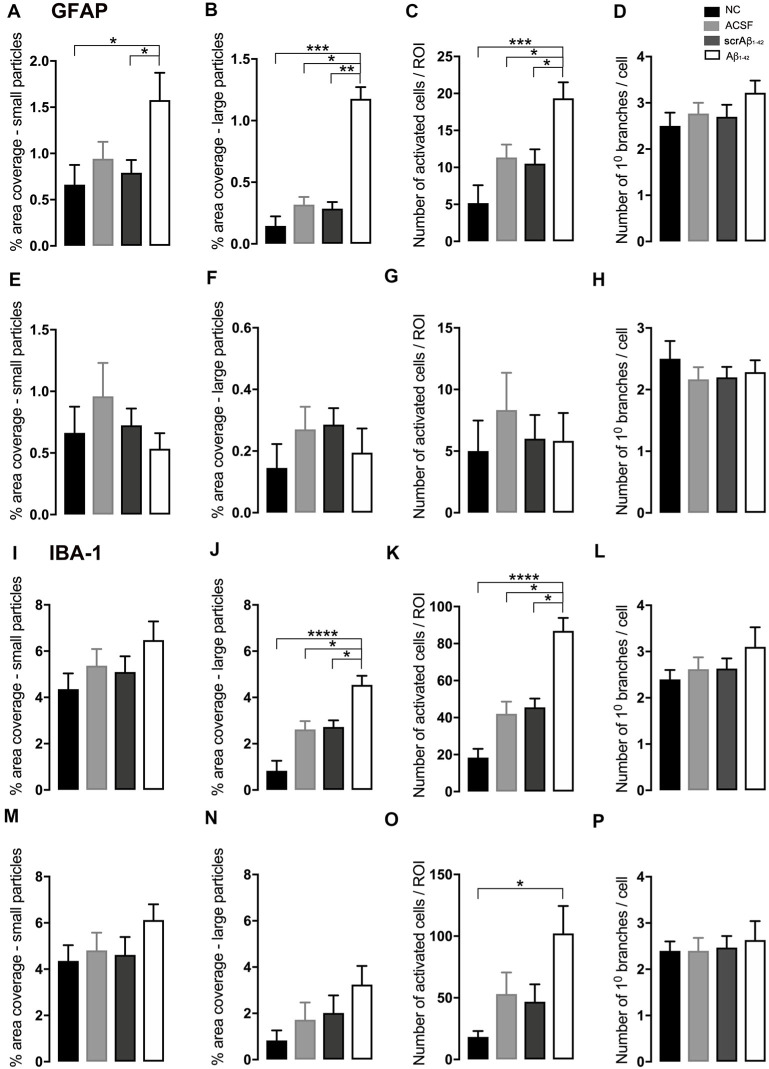
Graphs showing quantification of astrocytic and microglial morphology at the injection site **(A–D,I–L)** and a location adjacent to the injection site **(E–H,M–P)** in the CA1 hippocampal region 30 days post-injection in NC, ACSF-, scrAβ_1–42_- and Aβ_1–42_-injected mice. Data expressed as mean ± SEM (Kruskal–Wallis test; *p < 0.05, **p < 0.01, ***p < 0.001, ****p < 0.0001, *n* = 6).

In addition, astrogliosis was observed at the injection site by 30 days after the Aβ_1–42_ injection, as indicated by the significant increase in GFAP labeling in the str. oriens, str. pyramidale and str. radiatum in the Aβ_1–42_-injected mice in comparison with the NC (*p* < 0.0001), ACSF- (*p* = 0.0168; *p* = 0.0439; *p* = 0.0058) and scrAβ_1–42_-injected mice (*p* = 0.0168; *p* = 0.0057; *p* = 0.044; Figure 5IIIB). The ACSF- and scrAβ_1–42_-injected mice also displayed significantly higher GFAP immunoreactivity than the NC mice in the str. oriens (*p* = 0.0168; *p* = 0.0168), str. pyramidale (*p* = 0.0057; *p* = 0.0439) and str. radiatum (*p* = 0.044; *p* = 0.0058; Figure 5IIIB). Aβ_1–42_-injected mice showed an increased area coverage by activated cells (*p* = 0.0001 vs. NC; *p* = 0.0151 vs. ACSF; *p* = 0.0095 vs. scrAβ_1–42_), a greater number of astrocytes with reactive morphology (*p* = 0.0005 vs. NC; *p* = 0.049 vs. ACSF; *p* = 0.037 vs. scrAβ_1–42_), increased area coverage by astrocytic processes (*p* = 0.0128 vs. NC; *p* = 0.1651 vs. ACSF; *p* = 0.0455 vs. scrAβ_1–42_) but the number of primary branches did not change (Figures 6A–D).

Likewise, localized microgliosis, as indicated by the increase in Iba-1 density, in the str. oriens, str. pyramidale and str. radiatum was significantly increased in the Aβ_1–42_-injected mice compared with the NC (*p* < 0.0001), ACSF- (*p* = 0.0229; *p* = 0.0058; *p* = 0.0052) and scrAβ_1–42_-injected mice (*p* = 0.0124; *p* = 0.044; *p* = 0.0475; Figure 5IIIC). The ACSF- and scrAβ_1–42_-injected mice also displayed higher Iba-1 levels than the NC mice in the str. oriens (*p* = 0.0124; *p* = 0.0229), str. pyramidale (*p* = 0.044; *p* = 0.0058) and str. radiatum (*p* = 0.0475; 0.0052) of the CA1 region (Figure 5IIIC). Aβ_1–42_-injected mice displayed an increased area coverage by activated microglia (*p* < 0.0001 vs. NC; *p* = 0.0269 vs. ACSF; *p* = 0.049 vs. scrAβ_1–42_) and a greater number of microglia with reactive morphology (*p* < 0.0001 vs. NC; *p* = 0.0151 vs. ACSF; *p* = 0.0261 vs. scrAβ_1–42_) but the number of primary branches and the area covered by microglial processes did not change (Figures 6I–L).

Localized changes in vascular markers at the injection site were also observed in Aβ_1–42_-injected mice by 30 days after the injection as compared to the controls. CD31 levels were significantly higher in the Aβ_1–42_-injected mice than in the NC mice in the str. oriens, str. pyramidale, and str. radiatum of the CA1 region of the hippocampus (*p* = 0.0002; *p* < 0.0001; *p* < 0.0001; Figures 5IIn,IIID). The Aβ_1–42_-injected mice also displayed higher CD31 levels than the ACSF- and scrAβ_1–42_-injected mice in the str. pyramidale (*p* = 0.0135; *p* = 0.0025) and str. radiatum (*p* = 0.0162; *p* = 0.0042) of the CA1 region of the hippocampus (Figure 5IIID).

**Figure 7:**
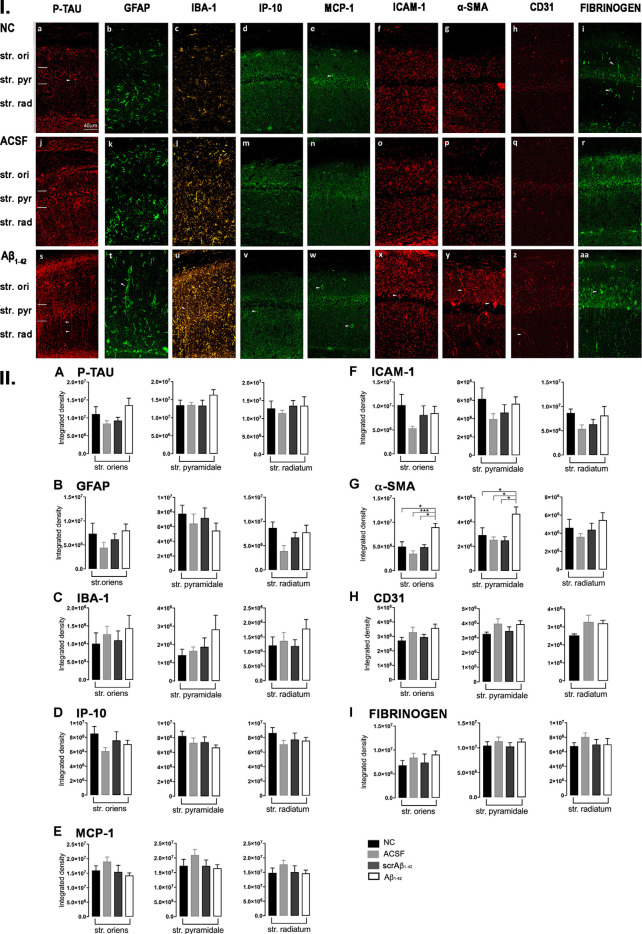
**Panel I:** Representative images showing immunolabeling of p-tau, GFAP, Iba-1, IP-10, MCP-1, ICAM-1, a-SMA, CD31 and fibrinogen at a location adjacent to the injection site, 30 days post- injection in NC **(a–i)**, ACSF-injected **(j–r)** and Aβ_1–42_-injected **(s-aa)** mice. **Panel II:** Graphs showing quantification at a location adjacent to the injection site in the CA1 hippocampal region of p-tau, GFAP, Iba-1, IP-10, MCP-1, ICAM-1, α-SMA, CD31 and fibrinogen immunolabeling density, 30 days post injection in NC, ACSF-, scrβ_1–42_- and Aβ_1–42_-injected mice. Data expressed as mean SEM (Kruskall-Wallis test; *p < 0.05; ***p < 0.001, *n* = 6).

In addition to the vascular changes found at the injection site, 30 days post-injection, there was a significant increase in α-SMA density at a location adjacent to the injection site in Aβ_1–42_-injected mice compared with NC, ACSF and scrAβ_1–42_-injected mice in the str. oriens (*p* = 0.0180 vs. NC; *p* = 0.0008 vs. ACSF; *p* = 0.0143 vs. scrAβ_1–42_) and str. pyramidale (*p* = 0.0241; vs. NC; *p* = 0.0246 vs. ACSF; *p* = 0.0136 vs. scrAβ_1–42_; Figures 7Iy,IIG). Although we did not find any other significant Aβ_1–42_-induced effects at the location adjacent to the injection site for p-tau, Iba-1, IP-10, MCP-1, ICAM-1, CD31, and fibrinogen (Figures 7I;IIA,D,E,F,H,I), Aβ_1–42_-injected mice showed a non-significant trend towards increased p-tau in the str. oriens of the CA1 hippocampal region (Figure 7Is). Astrocyte morphology did not differ from controls in Aβ_1–42_-injected mice (Figures 6E–H) but activated microglia numbers were slightly increased compared with NC (*p* = 0.0019), ACSF- (*p* = 0.1208) and scrAβ_1–42_-injected mice (*p* = 0.0864; Figures 6M–P).

**Figure 8:**
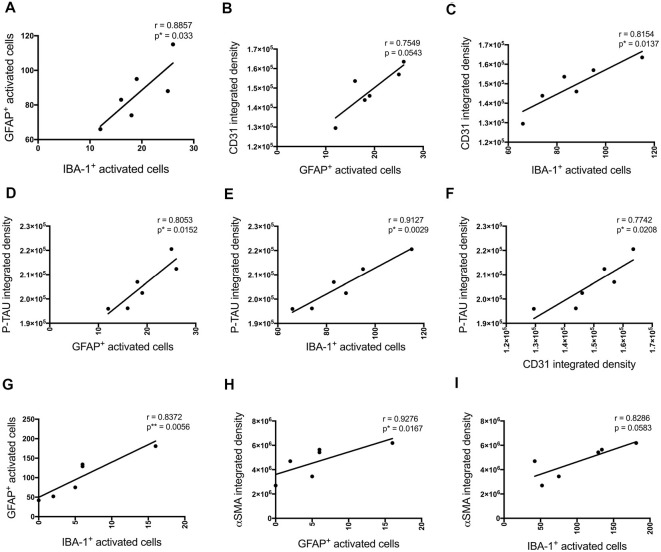
Correlation between the number of activated astrocytes and microglia, p-tau, and vascular markers at the injection site **(A–F)** and at a location adjacent to the injection site **(G–I)** in the CA1 hippocampal region 30 days post-injection in NC, ACSF-, scrAβ_1–42_- and Aβ_1–42_-injected mice. This relationship is represented by a Spearman’s *r*-value (*p < 0.05, **p < 0.01, *n* = 6).

P-tau, some inflammatory- (number of GFAP and IBA-1 positive activated cells) and vascular (CD31 and α-SMA density) pathology markers showed multiple positive correlations (Figure 8). Significant positive correlations were observed for most of these markers at the injection site and adjacent to the injection site as well. The number of activated GFAP positive cells positively correlates with the number of activated microglia (*r* = 0.8857, *p* = 0.033 injection site; *r* = 0.8372, *p* = 0.0056 adjacent injection site), CD31 (*r* = 0.7549, *p* = 0.0543), p-tau (*r* = 0.8053, *p* = 0.0152) and α-SMA (*r* = 0.9276, *p* = 0.0167 adjacent injection site) integrated density (Figures 8A,B,D,G,H). The number of IBA-1 positive activated cells also correlates with CD31 (*r* = 0.8154, *p* = 0.0137) and p-tau (*r* = 0.9127, *p* = 0.0029) integrated density (Figures 8C,E).

### Aβ_**1–42**_-Induced Behavioral and Cognitive Changes

**Figure 9:**
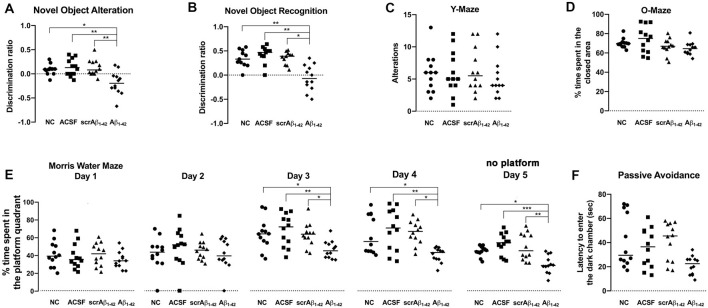
Aβ_1–42_-injected mice showed long-term spatial memory impairment revealed by the novel object alteration (**A**, performed on days 7, 8), novel object recognition (**B**, performed on days 11, 12), and Morris Water Maze (**E**, performed on days 20–24) tests. Short-term spatial memory assessed by the Y maze showed no significant difference in Aβ_1–42_-injected mice compared with controls (**C**, performed on day 15). Non-spatial memory remained unchanged in Aβ_1–42_-injected mice compared with controls (**F**, performed on day 27), and Aβ_1–42_ and/or the injection itself did not produce any anxiogenic effect (**D**, performed on day 17). Scatter plot showing all of the data values used with the median (Kruskal-Wallis test; *p < 0.05; **p < 0.01, ***p < 0.001, *n* = 12).

To elucidate the long-lasting effect of Aβ_1–42_ treatment on cognitive function at the 7–30-day time points the NOA, NOR and MWM tests for long-term spatial-memory were performed, as well as the YM test for short-term spatial memory, the passive avoidance test for short-term non-spatial memory, and the OM test as a measurement of the anxiety levels in the mice (Figures 9A–F).

According to the results of NOA (Figure 9A), the Aβ_1–42_-injected mice showed significant cognitive deterioration in long-term spatial memory as compared with the NC (*p* = 0.0308), ACSF- (*p* = 0.0082), and scrAβ_1–42_-injected mice (*p* = 0.0089), with significantly lower DR at 8 days post-injection (Figure 9A).

The Aβ_1–42_-injected mice also showed significant cognitive deterioration in long-term spatial memory as compared with the NC (*p* = 0.0099), ACSF- (*p* = 0.0003), and scrAβ_1–42_-injected mice (*p* = 0.0127) according to the results of the NOR test at 12 days post-injection (Figure 9B).

According to the MWM test, the Aβ_1–42_-injected mice also showed significant long-term spatial memory impairment when compared with the NC, ACSF- and scrAβ_1–42_-injected mice (Figure 9E). During day 1 and 2 of the MWM test (20 and 21 days post-injection), the mice from the three groups spent a similar amount of time in the platform quadrant of the maze, but significant differences were found between the NC, ACSF- and scrAβ_1–42_-injected mice and the Aβ_1–42_-injected mice during day 3 and 4 of the experiment: the Aβ_1–42_-injected mice spent significantly less time in the platform quadrant than the NC (*p* = 0.0401; *p* = 0.0498), ACSF-injected mice (*p* = 0.008; *p* = 0.0064) and the scrAβ_1–42_ mice (*p* = 0.0437; *p* = 0.0129 for day 3 and 4 respectively). During the fifth and last day of the test, when the platform was removed, the time spent by each group at the “platform quadrant” was also assessed. The Aβ_1–42_-injected mice spent significantly less time in the platform quadrant on day 5, as compared with the NC (*p* = 0.0287), ACSF- (*p* = 0.0003) and scrAβ_1–42_-injected mice (*p* = 0.0072; Figure 9E). Therefore, data from the MWM test show that whereas the control mice showed successful learning from day 3, the Aβ_1–42_-injected mice presented with long-term spatial memory impairment which affected their performance on days 3 and 4, as well as the last day of the test (Figure 9E).

According to the findings of the YM test, there was no significant difference in short-term spatial memory between any of the treatment groups 15 days post-injection. This indicates that short-term spatial memory was not affected by Aβ_1–42_ injection. Alternatively, it is also possible that any effect on short-term spatial memory could not be detected by this test. However, although the performance of the mice was similar across all the groups, the Aβ_1–42_-injected mice showed a slightly decreased number of alternations as compared with the NC, ACSF- and scrAβ_1–42_-injected mice (Figure 9C).

The Aβ_1–42_-injected mice showed no significant difference in non-spatial memory performance as compared with the NC (*p* = 0.0739), ACSF- (*p* = 0.3766) and scrAβ_1–42_-injected mice (*p* = 0.0539) by 28 days post-injection (Figure 9F). In phase 3 of the passive avoidance test (post-shock 3 h), similar latency to enter the dark chamber was found in the control and Aβ_1–42_-injected mice (Figure 9F).

Since anxiety is likely to influence cognitive performance, the OM test was performed to determine whether the mice from the different treatment groups exhibited anxiety. The Aβ_1–42_-injected mice showed no significant difference in anxiety levels as compared with the NC, ACSF- and scrAβ_1–42_-injected mice (Figure 9D), as mice from all the groups were found to spend a similar amount of time in the closed (protected) arm of the O-maze apparatus. Thus, Aβ_1–42_ and/or the injection itself did not produce any anxiogenic effect (Figure 9D).

**Figure 10:**
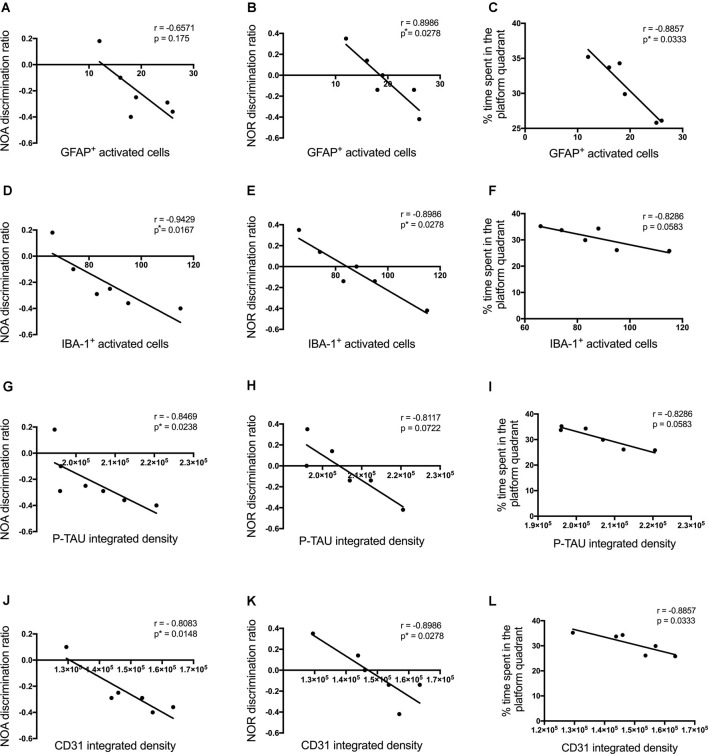
Correlation between the number of activated astrocytes and microglia **(A–F)**, p-tau **(G–I)**, CD31 **(J–L)**at the injection site in the CA1 hippocampal region, and behavioral scores 30 days post-injection in NC, ACSF-, scrAβ_1–42_- and Aβ_1–42_-injected mice. This relationship is represented by a Spearman’s *r*-value (*p < 0.05, *n* = 6).

Cognitive performance of Aβ_1–42_-injected mice after showed a negative correlation with p-tau density (NOA *r* = −0.8469, *p* = 0.0238; NOR *r* = −0.8117, *p* = 0.0722; MWM *r* = −0.8286, *p* = 0.0573; Figures 10G–I), the number of activated astrocytes (NOA *r* = −0.6571, *p* = 0.175; NOR *r* = −0.8986, *p* = 0.0278; MWM *r* = −0.8857, *p* = 0.0333; Figures 10A–C) and microglia (NOA *r* = −0.9429, *p* = 0.0167; NOR *r* = −0.8986, *p* = 0.0278; MWM *r* = −0.8286, *p* = 0.0583; Figures 10D–F) and CD31 density (NOA *r* = −0.8083, *p* = 0.0148; NOR *r* = −0.8986, *p* = 0.0278; MWM *r* = −0.8857, *p* = 0.0333; Figures 10J–L), but no significant correlations were observed with other inflammatory, vascular markers or neuronal loss.

## Discussion

The present study is an in-depth holistic molecular, cellular, and behavioral characterization of the acute and chronic effects of increased hippocampal Aβ_1–42_ concentration, achieved through bilateral intra-hippocampal Aβ_1–42_ injection in mice. We report that a single hippocampal Aβ_1–42_ injection resulted in localized layer-specific alterations in the abundance of inflammatory- and vascular markers and phosphorylated tau, at the injection site. At all the time points examined post-injection (7–30 days) significant long-term spatial memory impairments were found in Aβ_1–42_-injected mice compared to controls along with a significant neuronal cell loss in the str. pyramidale of the CA1 region. Our data suggest that inflammation and vascular disruption along with the local tau pathology observed in the CA1 region of the hippocampus might represent a relevant, complex, and interactive combination of leading factors that synergistically contribute to the hippocampus-dependent spatial memory impairment observed in the Aβ_1–42_-injected mice.

### Pyramidal Cell Loss in the CA1 Hippocampal Region of Aβ_**1–42**_-Injected Mice

We found a significant Aβ_1–42_ mediated pyramidal cell loss by 7 days in the str. pyramidale of the CA1 region of the hippocampus despite finding of an earlier study that hippocampal cell loss was not observed until 14 and 28 days, and not at 7 days (Takuma et al., 2004). This delayed response might be explained if there was a lower concentration of misfolded Aβ, and thereby fewer aggregates, perhaps because the less aggregation-prone Aβ_1–40_ was used. Although extrapolation of findings from rodent models to human AD must be made with caution, the excitatory pyramidal cell loss observed here does occur in late-stage AD in humans (Rossler et al., 2002) and, was at a relatively late outcome in our study as well.

The neurotoxic effect induced by high concentrations of Aβ_1–42_ observed in this study likely arose from a combination of factors, including damage from oxidative stress (Behl et al., 1995), activation of glial cells (Farfara et al., 2008), changes in intracellular Ca^2+^ concentrations and mitochondrial dysfunction (Arbel-Ornath et al., 2017). Aβ-induced apoptosis is likely mediated by the caspase-3-apoptotic cascade (Takuma et al., 2004; Brouillette et al., 2012; Vinnakota et al., 2020). Aβ_1–42_-induced cell death might also result from oxidative stress and the generation of oxidative modifications to lipid and protein in the cell—the presence of intracerebral Aβ_1–42_ in rats is associated with increased levels of oxidized proteins in the rat hippocampus (Boyd-Kimball et al., 2005). Aβ_1–42_-induced activation of glial cells and the generation of pro-inflammatory cytokines may also contribute to neurodegeneration *in vivo* (Farfara et al., 2008). Cell loss induced by hippocampal injection of Aβ_1–42_ in mice has been prevented by the administration of transforming growth factor (TGF)-β1, an immunosuppressive cytokine, which prevents glial activation and accumulation of Aβ_1–42_ (Chen et al., 2015). In the present study, the significant local upregulation of activated microglia and reactive astrocytes that were observed in the Aβ_1–42_-injected mice might also partially be responsible for the neural cell loss.

### Tau Pathology-Related Changes in Aβ_**1–42**_- Injected Mice

We report locally increased tau hyperphosphorylation by 30 days after Aβ_1–42_ injection in the str. oriens, str. pyramidale, and str. radiatum of the CA1 region of the hippocampus. Microglial activation seems to precede both tau hyperphosphorylation (Yoshiyama et al., 2007) and NFT formation (Maphis et al., 2016) and this might explain why, despite other changes, there is no p-tau pathology at day 3 after injection of Aβ_1–42_. The mechanism by which Aβ induces the activation of p-tau-dependent degeneration pathways in the cell has been widely investigated (Hurtado et al., 2010; Hu et al., 2014; Bennett et al., 2017). Hu et al. (2014) provide support for our conclusions as they demonstrated intra-hippocampal injection of aggregation susceptible Aβ_1–42_, but not Aβ_1–40_, in mice is responsible for the subsequent p-tau pathology of cell loss (Hu et al., 2014). Microglia may regulate tau phosphorylation through the microglial-specific fractalkine receptor (CX3CR1; Bhaskar et al., 2010). The ligand CX3CL1 is by contrast exclusively expressed by neurons and so neuron-microglia crosstalk likely precedes the p-tau pathology in AD. Indeed, disruption of the CX3CL1-CX3CR1 association has been shown to have a neuroprotective effect in a triple Tg AD mouse model(Fuhrmann et al., 2010).

Apart from contributions to both neuronal cell and synapse loss in the AD brain, p-tau pathology affects the generation of hippocampal theta oscillations, underlying the dysfunctional network circuitry in a triple Tg AD mouse model. A recent study reported that early tau pathology in the triple Tg AD mouse model resulted in a reduction in theta oscillations and overall excitability in the CA1 region of the hippocampus; perhaps as a compensatory mechanism for the prevention of Aβ-induced NMDA-mediated overexcitation (Mondragón-Rodríguez et al., 2018). Additionally, the accumulation of p-tau in parvalbumin-positive interneurons may also influence the changes in hippocampal activity and functionality (Soler et al., 2017). The tau pathology observed in the hippocampus and the activation of microglia might be one of the factors contributing to neurotoxicity and pyramidal cell loss in the CA1 region. While activation of microglia was observed further away from the injection site 3-day post Aβ_1–42_ injection, long-distance spreading from the injection site of the p-tau protein along the CA1 region was not found, indicating a longer-term activation of microglia might be required to trigger p-tau pathology. At day 30, p-tau correlates with the number of activated astrocytes and microglia at the injection site, but not at day 3 or adjacent to the injection site at day 30 where we did not observe activation of these cells.

### Inflammatory Changes in Aβ_**1–42**_-Injected Mice

Aβ_1–42_-induced inflammatory responses were confirmed by the local up-regulation of GFAP levels at day 3, as well as up-regulation of GFAP and Iba-1 levels by day 30, in Aβ_1–42_-injected mice compared with controls. Whereas control mice displayed sparse resting astrocytes, Aβ_1–42_-injected mice had an increased number of astrocytes with highly reactive morphologies depicted by numerous branching, elongated processes, and hypertrophic cell bodies. Astrogliosis, observable at day 3 and still present by day 30, occurred in the absence of significant increases in the immunoreactivity of the chemotactic factors IP-10 and MCP-1. Some studies have shown MCP-1 labeling near senile Aβ plaques, as well as reactive astrocytes expressing IP-10 in AD (McLarnon, 2012). Therefore, we hypothesize that the local acute inflammation following a single Aβ_1–42_ injection might not be sufficient to trigger the detectable production of chemotactic factors in the mouse hippocampus, as chemotactic factors in AD have been observed only during chronic neuroinflammation (McLarnon, 2012).

The activation of astrocytes seen on day 3 at the injection site was not maintained at an adjacent location until day 30. A previous study of Aβ-injection in a rat model showed that significantly elevated GFAP levels were observed 1 day after soluble Aβ injection, but not at day 30 (Weldon et al., 1998). We hypothesize that the system might be self-regulating with compensatory mechanisms for the inflammatory process in the proximity of the injection site and that acute astrocyte activation after Aβ_1–42_ injection could be transient. Activated astrocytes surrounding and isolating Aβ aggregates might represent the beginning of the Aβ clearance. On the other hand, the observed microglia activation responses on day 3 and day 30 post-injection had also occurred, which implies a phagocytic role for the microglia after the Aβ_1–42_ injection. Relationships between Aβ, neurons, astrocytes, and microglia in the AD brain are complex and should be further investigated.

### Vascular Changes in Aβ_**1–42**_-Injected Mice

Aβ_1–42_-injected mice also displayed signs of early vascular dysfunction by day 3 as revealed by the up-regulation of the endothelial cell marker CD31 in the str. radiatum of the CA1 region of the hippocampus. Consistent with this, early endothelial cell dysfunction has already been seen in other mouse models of AD (Lee et al., 2018a,b) and also in AD patients (Kelleher and Soiza, 2013), suggesting early alterations in blood flow regulation and in BBB permeability. Aβ-mediated increase in reactive oxygen species generation could lead to endothelial cell dysfunction through the alteration of endothelial tight junctions (Carrano et al., 2011). The acute up-regulation of the CD31 levels seen in this study might play a role in counteracting early Aβ-mediated effects on the endothelial cell and/or be involved with an early inflammatory process around the BBB. At this stage, the beginning of a disruptive process might affect BBB integrity.

Importantly, the early vascular dysfunction seen in Aβ_1–42_-injected mice is maintained up to day 30, and at this time point, CD31 labeling intensity was increased in the str. pyramidale and str. radiatum of the CA1 region of the hippocampus. In contrast to our results, reduced CD31 density was observed in 9-month-old Tg APP mice (Lee et al., 2018b), and Religa and colleagues demonstrated an inverse correlation between the number of plaques and CD31-labeled vessel density, which indicates that Aβ_1–42_ destroys the integrity of the BBB. They further suggested that Aβ_1–42_ led to apoptosis of endothelial and smooth muscle cells in AD patients and Tg TCRND8 APP mice (Religa et al., 2013). This apparent conflict with our findings might be resolved if the localized up-regulation of CD31 we observed is a compensatory molecular mechanism to re-establish homeostasis of the BBB, and to compensate for the loss of endothelial cells in cerebral capillaries during AD. Interestingly, tau overexpression affected endothelial cell functionality, as well as inducing vascular remodeling in a Tg AD mouse model (Bennett et al., 2018). Thus, local up-regulation of p-tau levels at the injection site in the present study may contribute to the dysfunction of endothelial cells in the BBB of Aβ_1–42_-injected mice. Indeed, p-tau levels show a positive correlation with CD31 levels on day 30 after Aβ_1–42_ injection.

Essential for the maintenance of vascular integrity in the brain, α-SMA has been extensively investigated in the context of AD pathogenesis. Here we report up-regulated α-SMA levels at the injection site in the CA1 region of the hippocampus in Aβ_1–42_-injected mice at day 30 post-injection. Furthermore, this up-regulation of α-SMA spread along the entire CA1 region. A lower degree of α-SMA immunostaining was found in the blood vessels of AD patients compared to controls (Ervin et al., 2004) but also increased expression of α-SMA in preclinical AD cases (Ervin et al., 2004). Consistent with our results, another AD mouse model was found to express high α-SMA immunostaining near Aβ plaques in the blood vessels in the cortex (Hutter-Schmid and Humpel, 2016). In preliminary studies, we have also detected an increase in α-SMA immunostaining in the middle temporal cortex of AD cases using tissue microarray methods (Austria et al., unpublished). It has been shown that smooth muscle cells undergo degeneration and atrophy during AD (Farkas and Luiten, 2001); therefore, the up-regulated α-SMA levels observed here might be an indicator of a systemic compensatory mechanism. Alternatively, since α-SMA regulates blood vessel contraction, its expression may be up-regulated to counteract any early dysfunction in blood flow occurring following Aβ_1–42_‘injection.

In the present study, up-regulated fibrinogen labeling at day 3 post-injection was also observed in the str. oriens of the CA1 region of the hippocampus. Three Tg AD mouse models, TgCRND8, PDAPP, and Tg2576, also have high levels of fibrinogen (Paul et al., 2007); confirming the contribution of fibrinogen to the pathology of AD, mostly *via* inflammatory processes. In agreement with this, in mouse models overexpressing APP in which fibrinogen was eliminated, microgliosis was found to be reduced (Paul et al., 2007). Fibrinogen infiltration and microglial reactivity have also been observed in Aβ_1–42_ intrahippocampal injected rodent brains and the human AD brain (Ryu and McLarnon, 2009). Indeed, increased fibrinogen density at day 3 shows a positive correlation with increased astrogliosis and microglia activation at day 3 after Aβ_1–42_ injection. Furthermore, increased fibrinogen deposition in Aβ_1–42_-injected mice might promote microvascular permeability through a negative effect on endothelial tight junction proteins (Tyagi et al., 2008), resulting in the accumulation of fibrinogen outside of circulation. Another possible mechanism by which fibrinogen could mediate BBB disruption is by affecting the accumulation and/or clearance process of Aβ in the vessels. Indeed, when fibrinogen levels were reduced in TgCRND8 AD mice, cerebral amyloid angiopathy was significantly diminished and reduced fibrinogen levels were linked to significant improvement in spatial memory (Cortes-Canteli et al., 2010). This finding indicates that one of the multiple factors associated with cognitive decline in the present AD mouse model could be the infiltration of fibrinogen into the hippocampal areas of the brain of Aβ_1–42_-injected mice, with the subsequent associated pathological events.

### Cognitive and Behavioral Changes in Aβ_**1–42**_-Injected Mice

A large number of Aβ-injected AD rodent models have demonstrated cognitive decline after infusion of the neurotoxic Aβ into rodent brains (Yamada et al., 1999; Nakamura et al., 2001; Tohda et al., 2003; Tsukuda et al., 2009; Takeda et al., 2014; Sadigh-Eteghad et al., 2015; Faucher et al., 2016). Spatial memory impairment is likely a result of the effects of increased Aβ concentrations at localized sites, confirmed by Aβ administration through both the i.c.v (Tsukuda et al., 2009; Kasza et al., 2017; Schmid et al., 2017) and hippocampal routes (Xuan et al., 2012).

Much evidence has shown that spatial memory impairment is caused by Aβ deposition and the subsequent synaptic dysfunction, among other Aβ-mediated effects, in the hippocampal area (Balducci et al., 2010). Exploration of novel objects is a critical approach to assess hippocampal-dependent spatial memory in AD rodent models. Our results demonstrate that Aβ_1–42_-injected mice showed hippocampal-dependent spatial memory impairment, as indicated by the results of the NOA, NOR and MWM tests on days 3 (Yeung et al., 2020b) and 30 post-injection, respectively. Previous studies have reported cognitive decline after Aβ infusion into rodent brains, assessed by NOR. Aβ_1–42_-mediated impairment of long-term spatial recognition memory was reported following a single injection of neurotoxic Aβ_1–42_ (i.c.v) into male C57BL/6 mice (Balducci et al., 2010). Takeda and colleagues found only very transient cognitive impairment after infusion of Aβ_1–42_ into the DG region of rats using the NOR test, and it was found to be associated with decreased long-term potentiation (LTP; Takeda et al., 2014). The short duration of the cognitive impairment indicates that the Aβ-mediated effect might vary among different areas of the hippocampus. Unlike this finding, our results demonstrated long-lasting Aβ_1–42_ effects on cognition, with the MWM test performed 20 days after the injection showing long-term spatial memory impairment in Aβ_1–42_-injected mice.

The MWM test is one of the most robust and most popular cognitive tests to assess hippocampal-dependent spatial memory (Tsukuda et al., 2009; Xuan et al., 2012; Esfandiary et al., 2015). We demonstrated that Aβ_1–42_-injected mice spent significantly less time in the platform quadrant than control mice during the last 2 days of the MWM test. In agreement with our findings, Xuan and colleagues also reported spatial memory impairment (using the MWM test) following the injection of Aβ_1–40_ into the dentate gyrus of the hippocampus of rats (Xuan et al., 2012). They observed astrogliosis and microgliosis in the hippocampus of the Aβ_1–40_-injected rats, consistent with our results in Aβ_1–42_-injected mice. Cognitive deficits were also demonstrated in Aβ_25–35_-injected mice (i.c.v administration) by the MWM test. Esfandiary and colleagues also demonstrated spatial-memory impairment (assessed by the MWM test) in an AD mouse model in which Aβ_1–42_ was intra-hippocampally injected into the CA1 region of the mice. In agreement with our findings, this study failed to demonstrate impairment in non-spatial memory, according to assessment with the passive avoidance test (Esfandiary et al., 2015). Aβ_1–42_ injection (i.c.v) in rats also resulted in cognitive deficits according to the MWM test (Zhang et al., 2015).

We did not find significant short-term spatial memory deficits with the YM test in Aβ_1–42_-injected mice, although there was a trend towards decreased alternations. Huh et al. (2014) found the percentage of alternations in mice where the DG was intra-hippocampally injected with Aβ_1–42_ was significantly lower than in the control group. Other studies have confirmed this with Aβ_1–42_-i.c.v injected mice (Yan et al., 2001) and rats (Zhang et al., 2015). Thus, the YM test might be a valid method to assess cognitive impairment in short-term memory induced by Aβ_1–42_ in rodent models.

In our study, no significant deficits were observed in non-spatial memory in the Aβ_1–42_-injected mice, utilizing the passive avoidance test (Esfandiary et al., 2015). These findings imply that the hippocampus might not be overly involved with non-spatial memory processes (Cave and Squire, 1991) and that Aβ affects only hippocampal-dependent memory processes. The passive avoidance test is used to assess a type of contextual memory which partly involves processing in the CA3 region of the hippocampus (Daumas et al., 2004) but our model is based on a single Aβ_1–42_ injection into the CA1 region, and that the CA1 area might be more critical in recognizing the novelty or familiarity of an object rather than contextual related-memories (Nakazawa et al., 2004; Daumas et al., 2005). This type of non-spatial memory likely remains unaffected in our experiments. An earlier study found that soon after Aβ_1–42_ i.c.v injection (day 1 and 7), mice exhibited deteriorated long-term non-spatial memory (Yan et al., 2001). Differences in the injected brain area, along with slight variations in the behavioral task design, might be factors that can potentially contribute to the divergence of results to those found in the literature.

The observed short-term spatial memory deficits correlate with p-tau, inflammatory and vascular pathology. Cognitive performance of Aβ_1–42_-injected mice showed a negative correlation with p-tau density, the number of activated astrocytes and microglia, and CD31 density. Most likely all these pathological changes are contributing factors to the cognitive deficits observed in these mice along with other molecular and cellular deficits. Short-term spatial memory deficits at day 3 occur before any significant neuronal loss (Yeung et al., 2020a), suggesting that all these pathological changes are sufficient to impair neural activity and information processing. The hippocampal Aβ_1–42_ injection in our study likely results in dysfunctional neural networks within the CA1, CA3 and the dentate gyrus, as there are widespread and complex interconnections within these hippocampal regions (Amaral et al., 2007). Likewise, it is well known that tau and amyloid can propagate throughout synaptically connected networks in the hippocampus (Cirrito et al., 2005; de Calignon et al., 2012). The physiological changes induced directly by Aβ_1–42_ throughout the hippocampus and other brain areas have been extensively studied but the link of network dysfunction with the complex pathological and behavioral changes needs to be further explored in future experiments.

In summary, our study shows that a single Aβ injection can reproduce aspects of the molecular, cellular, and vascular changes occurring in the AD human brain and can lead to cognitive deficits. We have demonstrated that not only classic Aβ and tau pathology features of AD contribute to the cognitive decline, but that neuroinflammation and vascular pathology may also play a key role in hippocampal-memory and learning deficits in AD.

## Data Availability Statement

All datasets presented in this study are included in the article.

## Ethics Statement

The animal study was reviewed and approved by the University of Otago Animal Ethics Committee and the University of Auckland Animal Ethics Committee.

## Author Contributions

BC-F, TC, TP, SW, KP, and AK: performed research. BC-F, TC, TP, SW, KP, JB, and AK: analyzed data. BC-F, TC, WT, HW, RF, and AK: wrote the article. WT, MD, and AK: designed research. MD, RF, and AK: funding acquisition. AK: project administration. WT, HW, RF, and AK: supervision. All authors contributed to the article and approved the submitted version.

## Conflict of Interest

The authors declare that the research was conducted in the absence of any commercial or financial relationships that could be construed as a potential conflict of interest.

## Abbreviations

Aβ: β-amyloid
ACSF: artificial cerebrospinal fluid
AD: Alzheimer’s disease
BBB: blood-brain barrier
CA 1: cornu ammonis region 1
CX3CR1: CX3C chemokine receptor 1
DR: discrimination ratio
E/I: excitatory/inhibitory
FPLC: fast protein liquid chromatography
IBA-1: ionized calcium-binding adaptor molecule 1
ICV: intracerebroventricular
LTP: long-term potentiation
GABA_A_Rs: GABA type (A) receptors
GFAP: glial fibrillary acidic protein
IP-10: interferon-inducible protein 10
MAPT: microtubule-associated protein Tau
MBP: maltose-binding protein
MCP-1: monocyte chemotactic protein-1
MWM: Morris water maze
NC: naïve control
NeuN: neuronal nuclei
NFTs: neurofibrillary tangles
NOA: novel object alteration
NOR: novel object recognition
NVU: neurovascular unit
OM: O-maze
PBST: phosphate-buffered saline with Tween-20
PFA: paraformaldehyde
PC: pyramidal cell
PMSF: phenylmethanesulfonyl fluoride
RT: room temperature
Str: stratum
TBS: Tris-buffered saline
TBST: tris-buffered saline Tween-20
TFA: trifluoroacetic acid
Tg: transgenic
TGF-β1: transforming growth factor-β 1
TTB: 0.05 M TBS/0.3% Triton/0.25% BSA
YM: Y-maze
α-SMA: alpha-smooth muscle actin.
